# Postbiotics as potential new therapeutic agents for sepsis

**DOI:** 10.1093/burnst/tkad022

**Published:** 2023-06-15

**Authors:** Xiran Lou, Jinfang Xue, Ruifei Shao, Chunyan Mo, Fuping Wang, Guobing Chen

**Affiliations:** Medical School, Kunming University of Science and Technology, 727 Jingming South Road, Chenggong District, Kunming 650500, China; Medical School, Kunming University of Science and Technology, 727 Jingming South Road, Chenggong District, Kunming 650500, China; Medical School, Kunming University of Science and Technology, 727 Jingming South Road, Chenggong District, Kunming 650500, China; Medical School, Kunming University of Science and Technology, 727 Jingming South Road, Chenggong District, Kunming 650500, China; Department of Emergency Medicine, The First People's Hospital of Yunnan Province, 157 Jinbi Road, Xishan District, Kunming 650034, China; Department of Emergency Medicine, The First People's Hospital of Yunnan Province, 157 Jinbi Road, Xishan District, Kunming 650034, China

**Keywords:** Sepsis, Gut microbiota, Dysbiosis, Postbiotics

## Abstract

Sepsis is the main cause of death in critically ill patients and gut microbiota dysbiosis plays a crucial role in sepsis. On the one hand, sepsis leads to the destruction of gut microbiota and induces and aggravates terminal organ dysfunction. On the other hand, the activation of pathogenic gut flora and the reduction in beneficial microbial products increase the susceptibility of the host to sepsis. Although probiotics or fecal microbiota transplantation preserve gut barrier function on multiple levels, their efficacy in sepsis with intestinal microbiota disruptions remains uncertain. Postbiotics consist of inactivated microbial cells or cell components. They possess antimicrobial, immunomodulatory, antioxidant and antiproliferative activities. Microbiota-targeted therapy strategies, such as postbiotics, may reduce the incidence of sepsis and improve the prognosis of patients with sepsis by regulating gut microbial metabolites, improving intestinal barrier integrity and changing the composition of the gut microbiota. They offer a variety of mechanisms and might even be superior to more conventional ‘biotics’ such as probiotics and prebiotics. In this review, we present an overview of the concept of postbiotics and summarize what is currently known about postbiotics and their prospective utility in sepsis therapy. Overall, postbiotics show promise as a viable adjunctive therapy option for sepsis.

Highlights

- The gut microbiota is involved in the development of sepsis and plays an important role in it.
- Postbiotics is the collective name given to probiotic bacteria and metabolites that can play the role of probiotics while avoiding many shortcomings of live bacteria and have been confirmed and applied in a variety of diseases.
- As a targeted treatment method of intestinal flora that has emerged in recent years, postbiotics have opened up a new direction for the treatment of sepsis.

## Background

The human digestive system is home to trillions of microorganisms, including fungi, viruses, bacteriophages, bacteria and archaea. They coexist and compete in the gut to maintain the balance between the host’s health and disease [1]. There are seven phyla of microorganisms in the gut: Firmicutes, Bacteroidetes, Actinobacteria, Proteobacteria, Fusobacteria, Cyanobacteria and Verrucomicrobia. Approximately 60–75% and 30–40% of the gut microbiota are from the phyla Firmicutes and Bacteroidetes, respectively [2,3]. The Firmicutes phylum is comprised primarily of gram-positive obligate or facultative anaerobic bacteria, such as *Clostridium spp*., *Enterococcus spp*. and *Lactobacillus spp.* [2,4]. The Bacteroidetes phylum is made up of primarily gram-negative anaerobic bacteria such as *Prevotella spp*. and *Bacteroides spp*. [2,4]. They help the body obtain energy from meals and decompose indigestible carbohydrates and proteins, digest and absorb nutrients, synthesize vitamins and promote host immunity [2]. The bacteria that live in a person’s digestive tract are referred to as commensals. Beneficial commensal microbes are capable of restoring the proper function of the intestinal barrier and exerting anti-inflammatory effects [5]. The microbiota affects human health and is related to some disorders.

Sepsis is a life-threatening organ dysfunction associated with a dysregulated host response to infection. It can lead to septic shock and multiple organ failure syndrome [6]. Current treatment emphasizes appropriate resuscitation with fluids, organ support, treating the infection with antibiotics and source control. Globally, there are >19 million cases of severe sepsis each year, leading to at least 5 million deaths [7]. There is no doubt that the global burden of morbidity and mortality caused by sepsis is substantial. During sepsis, the gut microbiome is severely changed. The ‘health-promoting’ resident gut flora (such as Bacteroides or Firmicutes) are significantly diminished, but potentially pathogenic microorganisms (such as Proteobacteria) increase [8]. The changed gut microbiota affects the inflammatory response and makes the intestinal barrier more permeable, which lets harmful germs enter the bloodstream and spread to other organs. This illustrates the importance of the gut microbiota in the development and progression of sepsis, suggesting a potential new therapeutic target for sepsis.

At present, there are many ways to treat disturbances of the gut flora in sepsis, such as selective digestive-tract decontamination, probiotics and fecal microbiota transplantation. However, there is growing evidence that the health advantages associated with probiotics are attributable not only to the presence of the live microbes themselves but also to the metabolites and secreted compounds produced by them [9]. These probiotic-derived metabolites are known as postbiotics. The International Scientific Association of Probiotics and Prebiotics established a definition of postbiotics in 2021, noting that postbiotics are ‘a preparation of inanimate microorganisms and/or their components that provides a health benefit to the host’ [10]. Postbiotics have great potential as a new-age product and have been used in food, pharmaceutical and clinical fields. Compared with probiotics, postbiotics have the advantages of long shelf-life and distinct molecular structure. It is important that postbiotics do not rely on living cells to accomplish probiotic functions, and can thus avoid the potential risks caused by live bacteria [11]. Although there is little research on postbiotics therapy for sepsis, we believe that this may be a feasible and effective adjunctive treatment for sepsis. In our previous research, we found that short-chain fatty acids (SCFAs) may reduce mortality in mice with sepsis by reducing serum proinflammatory cytokine levels, regulating the intestinal flora and improving the function of the intestinal barrier. The study provides a scientific basis for the development and utilization of postbiotics and provides new ideas for the prevention and treatment of sepsis [12].

## Review

### Gut microbiota and sepsis

#### Gut microbiota–sepsis interactions

Sepsis can result in intestinal dysfunction and disturbance of the intestinal microbiota, which can develop sequentially or simultaneously and influence one another [13]. However, the mechanism by which sepsis disrupts the gut microbiota remains unclear. Patients with sepsis may have altered intestinal microbiota due to mechanical ventilation, enteral or parenteral feeding, proton pump inhibitors, opioids, vasopressors, antibiotics and the sepsis stress state [14]. Inhibiting or killing dominant flora with several broad-spectrum antibiotics might enhance colonization by conditioning harmful bacteria and fungi, which in turn can cause opportunistic or secondary infections and exacerbate intestinal microbiome imbalances [15]. A prospective observational study confirmed that the use of antibiotics in the intensive care unit (ICU) greatly reduces the variety of intestinal microbiota [16].

Although antibiotics are the mainstay of treatment for sepsis patients, prolonged high-dose broad-spectrum antibiotic use can devastate gut flora, worsening the sepsis prognosis [17]. A study found that people who experienced antibiotic-induced dysbiosis while receiving an allogeneic bone marrow transplant had a 5- to 9-fold higher risk of bloodstream infection and sepsis [18]. Another retrospective cohort study of more than 10,000 elderly patients in the USA showed a >3-fold increase in the incidence of dysbiosis and subsequent hospitalizations for sepsis [19]. The results of our prior investigations demonstrated that germ-free animals during sepsis had increased bacterial transmission, inflammation, organ failure and mortality compared to healthy mice [12]. Similar effects of microbiome depletion and diversity loss on mortality have been evidenced in other sepsis experimental models [20,21]. During sepsis, the gut is typically dominated by a single bacterial genus, including multiple pathogenic and antibiotic-resistant bacteria, such as *Clostridia spp* and *Enterococcus spp.* Pathogenic bacteria release many toxic substances that kill intestinal epithelial cells and cause inflammatory reactions [22]. Additionally, intestinal permeability increases, mucosal tissue cells undergo ischemia and hypoxic damage, and bacteria and endotoxins from the intestine leak into the systemic circulation through the intestinal wall, enter the blood through the portal vein into distant organs and proliferate in large quantities, causing systemic infection, worsening sepsis and eventually leading to multiple organ failure syndrome [23].

#### Microbiota-targeted therapy in sepsis

**Figure 1:**
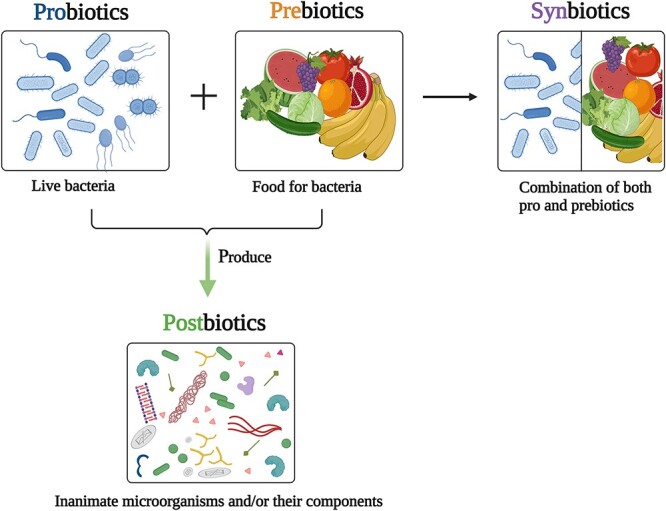
Probiotics, prebiotics, synbiotics and postbiotics. Probiotics are live strains of bacteria or specific living components of the microbiota, while prebiotics are substances that can be digested by the microbiota. Synbiotics are a combination of prebiotics and probiotics that have been used together. (Created with BioRender.com)

The significance of the gut microbiota as a homeostasis modulator that affects health and illness has emerged in recent years. Interventions that modify or are derived from the microbiota include probiotics, prebiotics, synbiotics and postbiotics (Figure 1). These therapies may prevent sepsis and improve patients’ prognoses [24].

Probiotics are living microorganisms that, when given to a host in sufficient quantities, can confer a health benefit on the host [25]. Prebiotics are substrates that are selectively utilized by host microorganisms, which then confer a health benefit on the host [26]. They are found naturally in foods such as sugar beets, asparagus, onions, chicory, barley, wheat and milk. The most prevalent types of prebiotics are fructo-oligosaccharides, galacto-oligosaccharides and *trans*-galacto-oligosaccharides [27]. Synbiotics are a mixture of live microorganisms and substrates that can be utilized selectively by microorganisms that are native and nonnative to the host [28]. Supplementation with high-quality probiotics, prebiotics and synbiotics can fight pathogenic bacteria and maintain the stability of gut flora, consequently increasing immune function and reducing disease incidence [29]. A meta-analysis showed that taking probiotics significantly lowers the risk of late-onset sepsis from 16.3% in the placebo group to 13.9% in the probiotic group [30]. In a cecal ligation and perforation mouse model, probiotic administration substantially reduced mortality by modulating microbiome makeup and metabolites [31]. Probiotics are usually well tolerated. They outcompete resident bacteria for nutrients and binding sites, produce bacteriocins that kill pathogens, increase IgA, improve mucosal immunity and reduce systemic inflammation [24]. The risk of neonatal sepsis and death has been shown to be dramatically reduced after the administration of a synbiotic containing *Lactobacillus plantarum* [32]. According to a meta-analysis of randomized controlled trials, synbiotics have been shown to be an effective and safe dietary therapy in lowering septic complications in critically sick patients [33].

However, despite extensive research on the effect of probiotics, prebiotics and synbiotics in protecting the host, the molecular mechanisms of action are complex and mostly unexplored. Bioavailability, infection risk and the potential transmission of an antibiotic-resistance gene are obstacles to its use in clinical therapies [34,35]. Barraud *et al*. conducted a randomized, double-blind, placebo-controlled trial study and found that nonsevere sepsis patients treated with probiotics showed a higher risk of death [36]. In another study, the use of probiotics increased bacterial translocation in patients with organ failure [37]. Probiotics were also tested in a large study that was conducted across multiple centers, and the results showed that they did not reduce the risk of ventilator-associated pneumonia or even other ICU inflammation [38]. Therefore, postbiotics, a group of preparations that act as a stand-in for probiotics, are garnering increasing attention.

### Postbiotics

#### The concept of postbiotics

**Figure 2:**
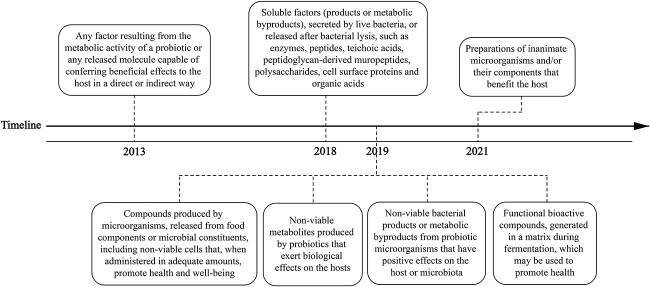
Timeline illustrating the main published definitions of postbiotics

Postbiotics, as proposed formally by Tsilingiri and Rescigno in 2013, are any factor that comes from the metabolic activity of a probiotic or any released molecule that can directly or indirectly help the host [39]. Since then, many scholars have given postbiotics several names and classifications. Commonly used labels for the inert components of probiotic cells have included ‘biogenic’, ‘cell-free supernatant’, ‘abiotic’, ‘metabiotic’, ‘pseudoprobiotic’, ‘ghost probiotic’ and ‘postbiotic’. The term ‘postbiotic’ is the one most commonly used in academic writing. In May 2021, the International Scientific Association of Probiotics and Prebiotics published a consensus declaration on postbiotics, clarifying the definition and scope of postbiotics for the first time. Postbiotics are a ‘preparation of inanimate microorganisms and/or their components that confers a health benefit on the host’ [10] (Figure 2).

#### The features and classification of postbiotics

Postbiotics include intentionally inactivated microbial cells with or without metabolites or cell components that contribute to health benefits but exclude pure microbial metabolites and vaccinations. In recent years, researchers have discovered many distinct postbiotic chemical types that originate from probiotic cells both outside and inside the host organism. Postbiotics have great potential in the future. The postbiotic components are composed of two parts. First, the components of beneficial bacteria are lipoic acid, phosphonic acid, peptidoglycans, cell surface proteins, polysaccharides, cell membrane proteins and extracellular polysaccharides. The second is the metabolites of beneficial bacteria: vitamins, lipids (butyrate, propionate, acetate, lactic acid etc.), enzymes, proteins (p40, p75 molecules), peptides, organic acids (propionic acid and 3-phenyl lactic acid etc.), SCFAs and intracellular polysaccharides [40,41]. The diversity of postbiotic components also translates to a variety of functions, including antibacteriostatic activity, immune regulation, antioxidant activity, liver protection, blood pressure-lowering activity, regulation of intestinal flora, and prevention and treatment of constipation, enteritis and other diseases [40–42]. In general, postbiotics have three advantages: higher safety, higher stability and a wider range of targets. In terms of safety, postbiotics are safer and also suitable for some special populations, such as newborns and sensitive people, since probiotics carry certain risks for these sensitive people. Postbiotics are also not inhibited by antibiotic interference, whereas probiotics are difficult to use simultaneously with antibiotics and carry a risk of transmitting resistance genes. In terms of stability, postbiotics are more stable and more resistant to oxygen, temperature and other environmental influences, and the active factors are not easily destroyed. Therefore, they have a longer shelf-life than live probiotics [43,44].

#### The use of postbiotics in disease

Postbiotics have limited evidence of therapeutic efficacy in human disease; thus, controlled clinical trials are needed. We reviewed the clinical postbiotic studies in adult and child cohorts discovered in the Cochrane central registration of controlled trials and a search of the MEDLINE database for randomized controlled trials, cohort studies and meta-analyses in adults and children. We discovered 24 different clinical trials involving postbiotics (Table 1). Seven studies focused on the use of postbiotics for the treatment of gut disorders, three of which focused on irritable bowel syndrome (IBS), two on *Helicobacter pylori* infection, one on obstructive jaundice and one on chronic diarrhea. In six studies, postbiotics were used to treat respiratory and pulmonary disorders. The remaining studies involved specific dermatitis, female menopause and obesity.

**Table 1:** Intervention studies of postbiotics in humans

**Postbiotics**	**Participants**	**Reference**
Nonviable probiotic lysate (BL) of *Escherichia coli* and *Enterococcus faecalis*	Patients with irritable bowel syndrome	Mack *et al*., 2022 [45]
Heat-inactivated *Bifidobacterium bifidum*	Patients with irritable bowel syndrome	Andresen *et al*., 2020 [46]
Inactivated *Lactobacillus*	Patients with diarrhea-predominant irritable bowel syndrome	Tarrerias *et al*., 2011 [61]
Nonviable *Lactobacillus reuteri*	*Helicobacter pylori*-positive patients	Yang *et al*., 2021 [48]
A lyophilized and inactivated culture of *Lactobacillus*	*H. pylori*-positive patients	Canducci *et al*., 2000 [47]
Inactivated *Lactobacillus plantarum* 299v	Patients with obstructive jaundice	Jones *et al*., 2013 [62]
Heat-killed *Lactobacillus acidophilus*	Patients with chronic diarrhea	Xiao *et al*., 2003 [63]
Live and dead cells of *Lactobacillus sakei* proBio65	Atopic dermatitis in children and adolescents	Rather *et al*., 2021 [49]
*Lactobacillus paracasei*	Atopic dermatitis in infants	D’Auria *et al*., 2021 [51]
*Lactobacillus rhamnosus* IDCC 3201 tyndallizate (RHT3201)	Atopic dermatitis	Jeong *et al*., 2020 [50]
Polyvalent mechanical bacterial lysate (PMBL)	Patients with moderate, severe or very severe COPD	Braido *et al*., 2015 [52]
Inactivated nontypeable *Haemophilus*	Patients with severe COPD	Tandon *et al*., 2010 [53]
Heat-killed *Mycobacterium manresensis*	Adults with or without latent TB infection	Montané *et al*., 2017 [54]
Polyvalent bacterial lysate and autovaccines	Patients with bacterial colonization of the nose and/or throat	Zagólski *et al*., 2015 [55]
Bacterial lysate Lantigen B	Patients with recurrent respiratory tract infections	Braido *et al*., 2014 [56]
Inactivated *Mycobacterium phlei*	Patients with moderate persistent asthma	Zhang *et al*., 2012 [64]
*Lactobacillus gasseri* CP2305	Mild menopausal symptoms in middle-aged women	Sawada *et al*., 2022 [57]
Heat-killed *Lactococcus lactis subsp.* cremoris H61	Young women	Takaragawa *et al*., 2022 [58]
Heat-inactivated *Bifidobacterium animalis subsp*. lactis CECT 8145, inulin, omega-3	Patients with abdominal obesity	Companys *et al*., 2022 [59]
Urolithin A (Mitopure)	Middle-aged adults	Singh *et al*., 2022 [60]
Resistant starch	Hypertension	Rhys-Jones *et al*., 2021 [65]
Heat-inactivated, washed and dried *Lactobacillus gasseri* CP2305	Young adults exposed to chronic stress	Nishida *et al*., 2019 [66]
Inactivated *Bacillus coagulans*	Responses to self-defense training in soldiers	Hoffman *et al*., 2019 [67]
Heat-inactivated *L. gasseri* CP2305	Stress responses in undergraduate medical students taking a cadaver course	Nishida *et al*., 2017 [68]

*BL* bacterial lysate, *PMBL* polyvalent mechanical bacterial lysate, *COPD* chronic obstructive pulmonary disease, *TB* tuberculosis

**Figure 3:**
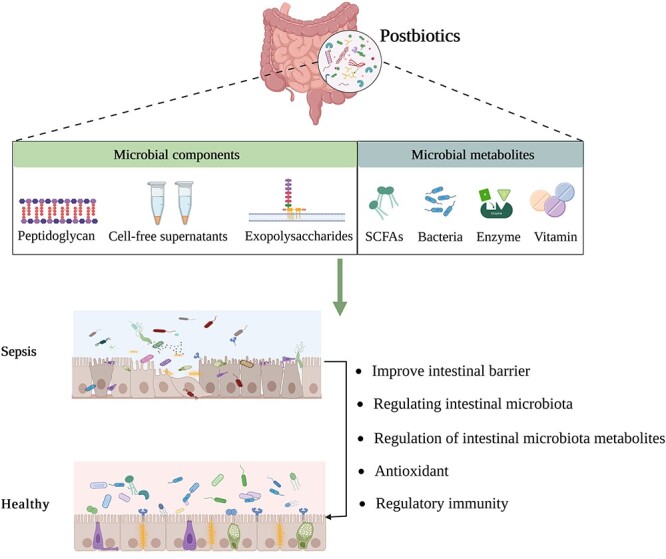
Postbiotics’ mechanism of action in sepsis. *SCFA* Short-chain fatty acids. (Created with BioRender.com)

In a study of 389 people with IBS who were given a nonviable probiotic lysate of *Escherichia coli* (DSM 17252) and *Enterococcus faecalis* (DSM 16440), the postbiotics significantly reduced IBS symptoms [45]. Similarly, heat-inactivated *Bifidobacterium bifidum* MIMBb75 improved pain in IBS patients compared to the control group [46]. Patients with *H. pylori* who were treated with clarithromycin, rabeprazole and amoxicillin had a greater rate of eradication when inactivated *Lactobacillus acidophilus* was also administered [47]. However, Yang *et al*. showed that adding nonviable *Lactobacillus reuteri* to triple therapy (esomeprazole amoxicillin and clarithromycin) did not increase the rate of eradication of *H. pylori* but did help to establish a beneficial microbial profile and decrease the occurrences of abdominal distention and diarrhea [48]. The oral administration of viable and nonviable *Lactobacillus sakei* proBio65 considerably ameliorated the symptoms of atopic dermatitis [49]. *Lactobacillus rhamnosus* IDCC 3201 tyndallizate also demonstrated therapeutic effects in children with moderate atopic dermatitis [50]. However, another study demonstrated that the use of the heat-inactivated probiotic *Lactobacillus paracasei* CBA L74 did not reduce the severity of atopic dermatitis [51]. Braido *et al*. recruited 287 patients with moderate to very severe chronic obstructive pulmonary disease and randomly allocated them to either a placebo group or a group treated with polyvalent mechanical bacterial lysates. When compared to the placebo group, those who were administered polyvalent mechanical bacterial lysate saw improvements in health and a reduction in symptoms such as fever, hospitalization and duration between exacerbations [52]. Another randomized, multicenter, double-blind, placebo-controlled trial indicated that treatment with HI-164OV for severe chronic obstructive pulmonary disease with frequent exacerbations was safe and helpful, especially in regard to reduction in severity parameters [53]. Furthermore, clinical research indicated that Nyaditum resae® (a galenic preparation of heat-killed *Mycobacterium manresensis*) can reduce the likelihood of developing active tuberculosis [54]. The colonization of *Haemophilus influenza* could be prevented by using polyvalent bacterial lysate [55]. Lantigen B, also a bacterial lysate, has been shown to decrease the frequency of acute episodes in people who suffer from recurrent respiratory tract infections [56]. Sawada *et al*. found that women aged 40–60 years who took CP2305 tablets for six menstrual cycles experienced a reduction in moderate menopausal symptoms [57]. Heat-killed *Lactococcus lactis* was demonstrated to improve iron absorption in young women [58]. Sticks of enriched seafood (heat-inactivated *Bifdobacterium animalis subsp.* lactis CECT 8145, inulin and omega-3) was shown to offer protection against the onset of type 2 diabetes and lower postprandial concentrations of atherogenic triglycerides in people with abdominal obesity [59]. In a randomized clinical experiment with middle-aged adults, the supplement urolithin A was found to boost both muscle strength and exercise performance as well as biomarkers of healthy mitochondria [60].

### The possible role of postbiotics in sepsis

There has not been a comprehensive assessment of postbiotics for the treatment or prevention of sepsis. To better understand the role that postbiotics (such as microbial structures, metabolites and inanimate microorganisms) play in sepsis, the major objective of this study was to summarize the current state of knowledge in this area (Figure 3).

#### SCFAs and sepsis

SCFAs are organic fatty acids with fewer than six carbon atoms in their molecular structure. Common SCFAs include acetate, propionate and butyrate, which are typically produced by beneficial bacteria in the intestine through the fermentation of dietary fiber [69]. The ratio of acetate to propionate to butyrate in the body of a healthy person is ~60 : 25 : 15 [70]. As the main source of energy for intestinal epithelial cells, SCFAs can help stabilize intestinal barrier function and fight inflammation. They can also enter the whole body through lymphatic circulation and humoral circulation and bind to their specific G-protein-coupled receptors (GPR43, GPR41 and GPR109a), which stops the expression of inflammatory factors controlled by histone deacetylase [69].

The function of SCFAs in sepsis has been gradually discovered in recent years. Studies have shown that taking soluble dietary fiber after gastrointestinal surgery greatly reduces the risk of sepsis in surgical patients [71]. One randomized controlled trial found that administering soluble dietary fiber to patients with severe pancreatitis reduced the incidence of enteral nutritional intolerance by a substantial amount [72]. Although there are few studies on the role of SCFAs in sepsis and related organ dysfunction, some meta-analyses have confirmed from different perspectives that soluble dietary fiber can improve intestinal microecology and indirectly reduce the risk of infection in severe patients [73,74]. Some studies comparing circulating and intestinal SCFA concentrations in sepsis patients using genomics have found that SCFA concentrations are closely related to prognosis [75,76]. In sepsis-associated encephalopathy, acetate and propionate levels declined dramatically, accompanied by gut microbiota dysbiosis and a decrease in SCFA-producing bacteria [77]. In addition, sodium butyrate was found to upregulate the level of the anti-inflammatory factor interleukin 10 (IL-10), which decreased the inflammatory response in mouse models of septic shock [78]. Okumura *et al*. showed that butyrate supplementation enhances intestinal barrier function and decreases septic mortality in rats [79]. SCFAs may play a key role in regulating inflammatory diseases by controlling the movement of immune cells to sites of inflammation, regulating the activity of immune cells and allowing the number of pathogens to decrease quickly [80].

#### Bacteriocins and sepsis

Bacteriocins are extracellular antimicrobial peptides (typically 10 kDa) produced in ribosomes by phylogenetically diverse bacteria and archaea. Probiotics produce a variety of bacteriocins that have bacteriostatic or bactericidal activity against bacteria of the same species or other unrelated genera and are widely used in food processing and fermentation as natural biological preservatives [40]. In addition, bacteriocins secreted by probiotics have a clinical role due to their demonstrated inhibitory ability against a number of antibiotic-resistant pathogens, such as *Mycobacterium tuberculosis* and *Listeria monocytogenes* infections [81]. Bacteriocins have the potential to suppress or kill drug-resistant organisms in contrast to traditional antibiotics due to their ability to disrupt bacterial cell membranes and cause the leakage of internal components [82]. The use of highly effective, narrow-spectrum bacteriocins as protein antibiotics is an alternative treatment approach for multidrug-resistant bacteria [83].

The spread of multidrug-resistant sepsis can be attributed to both the pervasive use of antibiotics and individual patient differences. Statistics showed that *Staphylococcus aureus* caused 120,000 cases of sepsis in the USA in 2017. Approximately 20,000 people die from sepsis, and the death rate from methicillin-resistant *S. aureus* (MRSA)-induced sepsis is high [84]. A study demonstrated that the novel leaderless bacteriocins Bacin A1, A2, A3 and A4 have powerful antimicrobial and antibiofilm activities against MRSA [85]. Additionally, sublancin (a glycosylated antimicrobial peptide generated by *Bacillus subtilis* 168) significantly reduced (*p* < 0.05) the bacterial burden and mortality of mice with an intraperitoneal MRSA infection [86]. Some research suggests that *Lactobacillus* is beneficial because it inhibits the growth of intestinal infections by increasing the production of antimicrobial molecules (designated bacteriocins) and triggering the release of immunoglobulin IgA [87]. Wang *et al*. discovered that treatment with *Companilactobacillus* crustorum MN047-derived bacteriocin prevented dysregulation of the gut microbiota, which was primarily manifested by a decrease in harmful bacteria and an increase in beneficial bacteria such as SCFA-producing bacteria, *Akkermansia* and *Blautia* [88]. Plantaricin A enhances the effectiveness of antibiotics by increasing the permeability of the outer membrane of bacteria. Meng *et al*. created a variety of plantaricin A1 analogs and showed that one of these antimicrobial peptides, OP4, increased the permeability of the bacterial outer membrane. In addition, OP4 effectively increased the efficacy of erythromycin and reduced the inflammatory reactions generated by an infection with *E. coli* [89]. The above results show that bacteriocins exert a variety of positive responses in the host, such as shaping bacterial community diversity and reducing the inflammatory response, and have the potential to replace traditional antibiotics as antibacterial drugs.

#### Enzymes and sepsis

Enzymes, which are produced by living cells, are proteins or RNAs that are extremely specific and catalytic for their substrates [90]. Enzymes produced by microorganisms catalyze a wide variety of reactions, including the hydrolysis of carbohydrates, the cleavage of proteins and the conversion of unsaturated fatty acids into saturated fatty acids, all of which improve food bioavailability and digestibility for human utilization [91,92].

Enzyme therapy has gradually become recognized and widely valued, and the clinical application of various enzyme preparations is becoming increasingly common. For example, trypsin, chymotrypsin and other similar enzymes can speed up the breakdown of proteins. This has been used in surgical expansion, cleaning pus-filled wounds and treating stereoscopic adhesions in the chest and abdomen [93,94]. Drugs such as plasmin, streptokinase and urokinase are used to dissolve blood clots and avoid their formation in the treatment of thrombophlebitis, myocardial infarction, pulmonary infarction and disseminated intravascular coagulation [95]. Yang *et al*. successfully redesigned a single-atom nanoenzyme (Cu-SAzyme) with an atomically dispersed Cu-N_4_ active site. The synthesized Cu-SAzyme showed good superoxide dismutase (SOD)-like activity, which significantly reduced systemic inflammation and reactive oxygen species (ROS)-induced multiorgan dysfunction, thereby significantly prolonging the survival time of mice with sepsis [96]. Cu-SAzyme has the potential to be an effective antioxidant and anti-inflammatory treatment for sepsis. Extracellular SOD2 induces neutrophil antithrombotic function and improves disseminated intravascular coagulation by preventing intraendothelial ROS accumulation and endothelial dysfunction [97]. Bao *et al*. used a lipopolysaccharide-induced sepsis mouse model and discovered that neutrophils inhibit sepsis-related coagulation dysfunction and enhance sepsis-related mouse survival via extracellular vesicles carrying SOD2 [97]. Moreover, extracellular SOD has been demonstrated to play a protective role in the early stages of sepsis development in rat models by decreasing peroxynitrite production in the renal arteries [98].

#### Vitamins and sepsis

Vitamins are a type of nutrient necessary for cellular metabolism [99]. Due to the inability of humans to synthesize most vitamins, they must be received from the diet, primarily from dairy and grain-based foods, fruits and vegetables. Vitamins are a class of trace organic substances that play an important role in growth, metabolism and development and are necessary for normal human and animal physiological functioning. They are classified into two groups: fat-soluble vitamins (A, D, E and K) and water-soluble vitamins (C and B vitamins) [100].

Vitamins play important roles in numerous biological processes that are relevant to sepsis. In adults and children alike, plasma vitamin deficiency is frequent in sepsis, and several observational and randomized controlled trials have linked vitamin therapy with better outcomes [101,102]. Water-soluble vitamin C is crucial for human health. High-dose intravenous vitamin C in adults with sepsis and acute respiratory distress syndrome reduced 28-day mortality and increased the number of days that patients spent outside of the ICU and hospital [101]. Another randomized controlled study showed that high dosages of vitamin C given to patients with sepsis improved tissue perfusion and oxygenation and alleviated subsequent organ failure [103]. In a mouse model of sepsis, intravenous vitamin C therapy protects the normal permeability of microcirculatory vascular endothelial cell membranes by suppressing peroxide formation. It also reduces cell edema and protects proteins from aberrant phosphorylation modification [104]. Kim *et al*. found that intravenous vitamin C administration decreased serum high-mobility group protein B1 levels and inhibited the release of the proinflammatory factors tumor necrosis factor-alpha (TNF-α), IL-1β, IL-6 and IL-8, thereby increasing the survival rate of septic mice [105]. In addition, vitamin D increases the expression of vitamin D-binding protein and antimicrobial peptides, which kill bacteria, deactivate toxins, reduce inflammation and inhibit endothelial cell death [106,107]. Serum vitamin D levels are significantly associated with infection risk and prognosis, and studies have shown that vitamin D deficiency or insufficiency is prevalent in patients with sepsis [108]. Patients with vitamin D deficiency had a substantially higher risk of dying while hospitalized, as shown in a retrospective study of 437 adults in the ICU [109]. A randomized controlled trial of vitamin D in 109 children with sepsis showed that it lowered inflammatory markers, cardiovascular organ failure scores and the risk of progressing to septic shock [110]. At present, vitamins have been widely used in clinical practice. Their strong antioxidant, anti-inflammatory, immunomodulating, microcirculation-protecting and immune-modulating properties, along with their good safety, make it possible to use them in patients with sepsis. In the future, it is anticipated that larger samples in prospective controlled clinical studies will confirm that vitamin supplementation can help prevent sepsis in vitamin-deficient patients and become an effective treatment for sepsis and septic shock.

#### Peptidoglycan and sepsis

Peptidoglycan, also known as murein, mucopeptide and glycopeptide, is a class of compact and solid macromolecular substances formed by crosslinking glycopeptides through a series of glycosidic bonds and peptide bonds, and its constituents are peptides and glycans [111]. Peptidoglycan is an important part of the bacterial cell wall, with the function of protecting cells from osmotic pressure and sustaining their normal morphology. It may be involved in a number of biological processes, such as boosting the immune system and fighting infections, tumors and allergies [112].

Studies have shown that peptidoglycan enters the host via oral or nongastrointestinal routes, enhancing the host’s immune surveillance function, boosting the production of various cytokines and antibodies by lymphocytes in immune organs and bolstering the activity of macrophages and natural killer cells to better regulate cells and fight infections [113]. Peptidoglycan from *L. acidophilus* inhibited lipopolysaccharides (LPS)-stimulated cyclooxygenase-2 and inducible nitric oxide synthase expression in RAW 264.7 cells [114]. Wu *et al*. reported that peptidoglycan decreased the production of LPS-induced inflammatory cytokines through inhibition of the toll-like receptor 4 (TLR-4) pathway [115]. In another study, nasal injection of *L. rhamnosus* CRL1505 peptidoglycan (PG05) improved not only the innate immune response but also the respiratory and systemic adaptive humoral response. PG05 boosted the T helper 2 (Th2) response, B-cell recovery, and anti-pneumococcal antibody concentration and activity [116]. In addition to immunomodulatory functions, researchers have extensively investigated the anticancer properties of peptidoglycan [117]. *Lactobacillus* peptidoglycan can trigger the production of TNF-α, which in turn binds to the Fas–Fas ligand system on target cells to suppress tumor cell growth and trigger programmed cell death. Furthermore, peptidoglycan can suppress the G1 to S phases of cancer cells, preventing DNA synthesis and ultimately leading to antitumor effects by preventing cancer cells from multiplying. According to recent research, active *Enterococci* express and release NlpC/p60 peptidoglycan hydrolase SagA and its homologs, which generate immunoreactive peptides (muropeptides) that stimulate an immune response to treatment via the peptidoglycan sensor nucleotide-binding oligomerization domain 2 (NOD2). Microbiota with unique peptidoglycan remodeling activity and muropeptide-based treatments have both been proposed as potential cancer immunotherapy enhancements and next-generation adjuvants [118]. Currently, only the immunomodulatory, antiproliferative or antitumor activity of peptidoglycan isolated from probiotics has been investigated, and the effects of peptidoglycan on sepsis and related dysfunction have received scant attention. Therefore, additional research is required to identify all potential sepsis-related signaling pathways.

#### Cell-free supernatants and sepsis

Bacterial cells are separated from their growth media using centrifugation and filtering. The resultant fluid is known as cell-free supernatant (CFS) [119]. It has been demonstrated to reduce inflammation, function as an antioxidant, fight germs and viruses, and inhibit tumor growth [41]. CFSs include biomolecular and active metabolites (carbon dioxide, organic acids, diacetylene, bacteriocin-like compounds etc.) that are typically secreted by lactic acid bacteria and yeasts [120].

Previous studies have shown that the CFSs of *L. acidophilus* and *L. casei* have anti-inflammatory and antioxidant effects on intestinal epithelial cells, macrophages and neutrophils by decreasing TNF-α release and boosting the secretion of the anti-inflammatory cytokine IL-10 [121,122]. Batista *et al*. discovered that CFSs reduced neutrophil cells entering the small intestine mucosa and improved intestinal epithelial architecture following 5-FU injury. These beneficial results were associated with elevated levels of the immunoregulatory cytokine IL-10 and epithelial barrier indicators and decreased levels of inflammatory markers [123]. Kareem *et al*. found that the CFSs of the *L. plantarum* RG11, RG14, RI11, UL4, TL1 and RS5 strains inhibited the growth of *L. monocytogenes* L-MS, *Salmonella enterica* S-1000, *E. coli* E-30 and vancomycin-resistant *Enterococci* [124]. Additionally, the CFSs of *L*. *rhamnosus* GG exhibited potent antibacterial activity against *E. coli* K1, inhibiting adhesion, invasion and translocation by stimulating mucin production and preserving intestinal barrier function [125]. Although the study of CFSs and sepsis has not been reported, the above results indicate that CFSs have a wide range of potential applications for regulating intestinal barrier function and ameliorating intestinal flora disorders.

#### Exopolysaccharides and sepsis

Exopolysaccharides (EPSs) are classified as either homopolysaccharide (dextran, levan) or heteropolysaccharide (kefiran), each of which is composed of two or more different types of monosaccharides [126]. EPSs are carbohydrate polymers found on the outer membranes of many different types of cells, including bacteria. They can be found in capsule form on the surface of the cell or secreted as a slimy substance into the surrounding environment [127].

*In vitro* and *in vivo* studies have associated EPS compounds from *Bifidobacterium, Lactobacillus, Leuconostoc* and *Bacillus* with health benefits [128,129]. The EPSs produced by *Bifidobacterium bifidum* and *Bifidobacterium longum* promote the growth of *Lactobacilli* and other beneficial anaerobic bacteria while inhibiting the growth of harmful bacteria [130]. Wang *et al*. have shown that the EPSs produced from *L. plantarum* JLK0142 can stimulate the immune system in cyclophosphamide-induced immunosuppressed animals and increase the immunomodulatory activity of RAW 264.7 cells [131]. One study showed that EPSs from *Streptococcus thermophilus* improved intestinal barrier function by decreasing proinflammatory cytokine production and increasing the expression of tight junction proteins (claudin-1, occludin and E-canherin) [132]. EPSs produced by *S. thermophilus* CRL1190 enhance the anti-inflammatory response by modulating the production of the cytokine IL-8 [133]. Furthermore, it has been shown that the EPSs from *Bacillus sp.* strain LBP2 can inhibit LPS-induced inflammation in macrophages by reducing the production of ROS and nuclear factor kappa B (NF-κB) activation [134]. A study by Maeda *et al*. found that an exopolysaccharide (kefiran) significantly impacted lipids, blood pressure, blood glucose and gastrointestinal symptoms such as bloating and gas. Kefiran has potential as a functional meal for the treatment and prevention of several illnesses [135].

### Current status and perspectives for clinical practice

The gastrointestinal tract is the first and most vulnerable organ to be affected by the pathological process of sepsis. Some clinical treatment methods, such as mechanical ventilation, vasoactive drugs and broad-spectrum antibiotics, can disrupt the intestinal flora and impair barrier function [12]. Probiotics and fecal microbiota transplantation are key research areas for treating intestinal flora disorders in sepsis and regulating the disease course. While they has some effects, there are numerous controversies. As living bacteria, their bioavailability in the intestine is difficult to measure, and it has been reported that resistance genes can be transmitted from donor to recipient via fecal microbiota transplantation [136]. Postbiotics are a class of probiotic products with probiotic effects that have multiple functions, including maintaining intestinal flora, protecting intestinal barrier function and regulating the immune system. Compared to live bacteria, their greatest advantage are their distinct chemical structure and high absorption and distribution rates. Postbiotics can perform the role of probiotics while avoiding many of the drawbacks of live bacteria, indicating a new treatment strategy for sepsis. Current research on postbiotics and sepsis is in its infancy, and a large number of systematic fundamental studies must be conducted in conjunction with late-stage clinical trials for confirmation. It is possible to determine the precise treatment of intestinal flora disorders in sepsis by systematically investigating the efficacy of postbiotics and integrating, optimizing and utilizing various probiotic components. The study of postbiotics in the treatment of sepsis must overcome many theoretical and technological problems, and their mechanism of action must be further studied, analyzed and summarized. To facilitate the identification and distribution of postbiotics, there needs to be a centralized reporting system set up where researchers can discuss the specifics of their postbiotic agents, including their ingredients, their inactivation techniques and the microorganisms used in their production. This will assist in combining the studys’ findings and accelerating the product’s introduction into clinical use. Ultimately, the efficacy and safety of postbiotics in patients with sepsis must be examined from a thorough pharmacologic standpoint and in larger randomized controlled trials.

## Conclusions

To our knowledge, this is the first review to synthesize the evidence on the impact of postbiotics on sepsis. Postbiotics offer an attractive alternative to probiotics since they are safe and stable and have a long shelf-life, making them easy to store and transport. Postbiotics can also be given to patients while they are receiving antibiotic treatment without compromising the treatment’s effectiveness. Therefore, the discovery of postbiotics and the continuation of research on them has led to a better understanding of how they fight diseases and paved the way for the creation of new medications that do not have living cells or physiological effects.

## Abbreviations

CFSs: Cell-free supernatants; EPSs: Exopolysaccharides; IBS: Irritable bowel syndrome; ICU: Intensive care unit; IL: Interleukin; MRSA: Methicillin-resistant *Staphylococcus aureus;* SCFAs: Short-chain fatty acids; SOD: Superoxide dismutase; ROS: Reactive oxygen species; TNF-α: Tumor necrosis factor α; LPS: Lipopolysaccharides; TLR-4: Toll-like receptor 4; Th2: T helper 2; NOD2: nucleotide-binding oligomerization domain 2; NF-κB: nuclear factor kappa B.

## Funding

This study was supported by the National Natural Science Foundation of China (No. 82160366), Yunnan Young and Middle-aged Academic and Technical Leaders Reserve Talent Project (No. C098-2060499), Yunnan Clinical Medical Center Open Project (No. 2021LCZXXF-HX03) and Yunnan Key Laboratory of Stomatology Open Project (No. 2022YNKQ004).

## Authors’ contributions

XL and GC designed and wrote the manuscript. JX and RS analyzed the feasibility of the article and searched the literature. CM and FW searched the literature and revised the article. All the authors have read and agreed to the published version of the manuscript.

## Conflict of interests

The authors declare that they have no competing interests.

## Data availability

Data is openly available in a public repository.
